# The importance of computed tomography of the chest in cases of suspected infection with nontuberculous mycobacteria (*Mycobacterium kansasii*)

**DOI:** 10.1590/0100-3984.2016.49.4e1

**Published:** 2016

**Authors:** Miriam Menna Barreto, Rosana Souza Rodrigues

**Affiliations:** 1 Radiology Department, Hospital Universitário Clementino Fraga Filho - Universidade Federal do Rio de Janeiro (HUCFF-UFRJ), Rio de Janeiro, RJ, Brazil. E-mail: miriam.menna@gmail.com.; 2 Radiology Department, Hospital Universitário Clementino Fraga Filho - Universidade Federal do Rio de Janeiro (HUCFF-UFRJ), D'Or Institute for Research and Education, Rio de Janeiro, RJ, Brazil. E-mail: rosana.souzarodrigues@gmail.com.

Imaging studies of patients with tuberculosis have been the subject of a number of recent publications in Brazil radiology literature^(1,5)^. Nontuberculous mycobacteria (NTM) are increasingly recognized as a major cause of lung infection in immunocompetent patients. Unlike *Mycobacterium tuberculosis*, that only humans are reservoirs, NTM are frequently isolated from environmental sources, such as water and soil; NTM infection can also evolve to severe lung disease, being a common cause of morbidity and mortality^(6-8)^. Lung disease secondary to NTM infection occurs primarily in elderly individuals, with or without comorbidities, and the leading causative agent is *Mycobacterium avium intracellulare*, followed by *Mycobacterium kansasii*^(9)^.

NTM diagnosis is difficult because clinical manifestations are nonspecific and isolation of bacteria in sputum or bronchoalveolar lavage fluid may only represent airway colonization. The American Thoracic Society^(10)^ established criteria for NTM diagnosis including clinical data, identification of the mycobacteria, and imaging findings. Therefore, knowledge of the radiological and tomographic aspects of NTM infection, including *M. kansasii*, plays an important role in the definitive diagnosis.

In the excellent study published in this issue of **Radiologia Brasileira**, Mogami et al.^(11)^ discuss the main computed tomography (CT) findings in 19 patients with pulmonary infection by *M. kansasii*, all confirmed by the established criteria. On CT scans, NTM-related pulmonary infection may present in one of the three forms^(12)^: 1 - the classic presentation, similar to tuberculosis; 2 - bronchiectatic; and 3 - hypersensitivity pneumonitis. The bronchiectatic form, common in middle-aged women, is more characteristic and presents as centrilobular nodules, with or without a tree-in-bud pattern, associated with cylindrical bronchiectasis, typically involving the middle lobe and lingula^(13)^. In the study conducted by Mogami et al.^(11)^ bronchiectasis occurred more often in the upper lobes, suggesting that radiologists should be aware of atypical manifestations of MTN infection.

Mogami et al.^(11)^ study is of great value, especially in Brazil that has high incidence of tuberculosis, and the differential diagnoses of NTM is relevant given that presumptive treatment with antituberculosis drugs is a common practice. Pulmonary CT manifestations of *M. kansasii* infection may be indistinguishable of *M. tuberculosis*, as demonstrated in this study. The authors concluded that presence of cavities and involvement of the small and large and airways (characterized by bronchiectasis and changes due to filling of the bronchioles) were common. The authors also highlighted other interesting finding: architectural distortion was found in almost 90% of the patients, which could be attributed to the high prevalence of tuberculosis in the studied population.

In conclusion, Mogami et al.^(11)^ reported chest CT findings that could help radiologists suspect of *M. kansasii* infection and include this condition in the differential diagnosis.
